# S-containing and Si-containing compounds as highly effective electrolyte additives for SiO_*x*_ -based anodes/NCM 811 cathodes in lithium ion cells

**DOI:** 10.1038/s41598-019-49568-1

**Published:** 2019-10-01

**Authors:** Fuqiang An, Hongliang Zhao, Weinan Zhou, Yonghong Ma, Ping Li

**Affiliations:** 1 Beijing University of Science and Technology, No. 30 Collage Road, Haidian District, Beijing China; 2 Shanxi Changzheng Power Technology Co., Ltd., Shanxi, China; 3 Idrivetech Automobile Co., Ltd. No. 2 Nanqi Road, ChangPing District, Beijing China

**Keywords:** Batteries, Batteries

## Abstract

Recently, high-energy density cells containing nickel-rich cathodes and silicon-based anodes have become a practical solution for increasing the driving range of electric vehicles. However, their long-term durability and storage performance is comparatively poor because of the unstable cathode-electrolyte-interphase (CEI) of the high-reactivity cathode and the continuous solid-electrolyte-interphase (SEI) growth. In this work, we study several electrolyte systems consisting of various additives, such as S-containing (1,3,2-dioxathiolane 2,2-dioxide (DTD), DTD + prop-1-ene-1,3-sultone (PES), methylene methanedisulfonate (MMDS)) and Si-containing (tris(trimethylsilyl) phosphate (TTSP) and tris(trimethylsilyl) borate (TMSB)) compounds, in comparison to the baseline electrolyte (BL = 1.0 M LiPF_6_ + 3:5:2 w-w:w EC: EMC: DEC + 0.5 wt% lithium difluoro(oxalato)borate (LiDFOB) + 2 wt% lithium bis(fluorosulfonyl)imide (LiFSI) + 2 wt% fluoroethylene carbonate (FEC) + 1 wt% 1,3-propane sultone (PS)). Generally, electrolytes with Si-containing additives, particularly BL + 0.5% TTSP, show a lower impedance increase in the full cell, better beginning-of-life (BOL) performance, less reversible capacity loss through long-term cycles and better storage at elevated temperatures than do electrolytes with S-containing additives. On the contrary, electrolytes with S-containing additives exhibit the advantage of low SEI impedance but yield a worse performance in the full cell than do those with Si-containing additives. The difference between two types of additives is attributed to the distinct function of the electrodes, which is characterized by cyclic voltammetry (CV), electrochemical impedance spectroscopy (EIS) and X-ray photoelectron spectroscopy (XPS), which was performed on full cells and half cells with fresh and harvested electrodes.

## Introduction

Lithium ion batteries (LIBs) are now widely applied in electric vehicles, owing to their higher energy density relative to other energy storage devices^1–4^. This advanced performance promotes their commercial application in electric vehicles (EVs).

To increase the energy density of LIBs, nickel manganese cobalt oxides (LiNi_*x*_Mn_*y*_Co_*z*_O_2_, abbreviated as NCM) have been widely used as a good candidate cathode material^5–7^. Recently, the gravimetric energy of lithium ion cells has increased from 200 Wh/kg to 240 Wh/kg due to the application of LiNi_0.5_Mn_0.3_Co_0.2_O_2_ (NMC532) and LiNi_0.6_Mn_0.2_Co_0.2_O_2_ (NMC622). Meanwhile, LiNi_0.8_Mn_0.1_Co_0.1_O_2_ (NMC811) has been widely studied and gradually industrialized. Some scientific questions should be addressed before its marketization, such as through examining the rapid growth of impedance, gas evolution, and electrolyte depletion upon repeat charge-discharge processes^8,9^.

Much effort has been made to improve the electrochemical performance of NCM systems. One method is to develop a custom electrolyte system to overcome the depletion of NCM. However, traditional electrolyte systems suitable for conventional layered material cannot completely avoid continuous side reactions through passivating the cathode-electrolyte-interphase of Ni-rich NCM. Therefore, more effective additives have been reported, and 1,3-propane sultone (PS) is one of most useful early-reported additives for layered martials because it induces low impedance of the cathode-electrolyte-interphase (CEI), which is the reason behind the obvious improvement of storage and cycle performances^10^. However, the reduction of PS leads to a large impedance of the solid-electrolyte-interphase (SEI) film. Recently, phosphites (P(OR)_3_), phosphates (OP(OR)_3_), borates (B(OR)_3_), and boroxane (*c*-B_3_O_3_(OR)_3_) derivatives, such as tris(trimethylsilyl) phosphite (TTSPi or TMSPi)^11–13^, tris(trimethylsilyl) phosphate (TTSP or TMSP)^14–16^, and tris(trimethylsilyl) borate (TMSB)^17–22^, have been developed as cathode-protective agents, especially for application with nickel-rich layered materials. However, there is a controversy over whether the protective function arises from the scavenging of HF from LiPF_6_ hydrolysis through the O-Si bond-breaking pathway^11,12^ or from binding of the reaction centres of the layered material to inhibit oxygen removal from the surface by the reaction products^13^. Nevertheless, the function of the additives is widely acknowledged to be preventing the dissolution of transition metal from the cathode, thus forming a stable CEI with a lower impedance increase^11–13^.

Silicon and silicon oxide, of which the theoretic capacities are 3579 mAh/g (Li15Si4) and 1800 mAh/g, show large volume changes, leading to the pulverization or electric isolation of particles and continuous SEI growth during repeated charge-discharge processes^23–25^. Silicon-based graphite-carbon composites, such as silicon oxide/carbon/graphite (abbreviated as SiOx/C/Gr) and silicon/carbon/graphite (abbreviated as Si/C/Gr), are promising commercial anode materials, because these materials show the balanced advantages of graphite and silicon or silicon oxide without much depletion^26–28^. However, the amorphous Si is ball-milled and dispersed in the framework of SiO2 or directly encapsulated by amorphous carbon, and commercial silicon-based materials such as SiOx/C/Gr and Si/C/Gr have many inevitable parasitic reactions with electrolytes and exhibit large volume expanses towards graphite^29^.

Similar to case for efforts devoted to cathode materials focusing on the CEI, it is still a challenge to tailor the electrolyte for stabilizing the SEI film in silicon-based composite anode materials. FEC is an essential additive in silicon-based negative electrodes, which obviously enhances the stability of the solid-electrolyte-interphase (SEI)^30–32^. FEC is a useful negative SEI-forming additive for Si and graphite anodes, and it has been reported that FEC is more suitable for Si anode, forming a more stable SEI than that formed with graphite and leading to the desired excellent performance of Si-based composite anodes^30^. Second, FEC is oxidized on the positive electrode, forming a LiF-rich compound to stabilize the CEI^31^. Moreover, it is noteworthy that the FEC content must be carefully adjusted according to the Si content in the composite anode material. It has been reported that worse performance at elevated temperatures was caused by excessive FEC^33,34^ and that sharp deterioration during cycle tests resulted from insufficient FEC^35^. Cyclic sulfate derivatives, such as 1,3,2-dioxathiolane 2,2-dioxide (DTD)^36–38^, prop-1-ene-1,3-sultone (PES)^39–43^, and methylene methanedisulfonate (MMDS)^38,44–46^, have been proposed as solid-electrolyte-interface (SEI)-forming additives for application on negative electrodes. DTD decreases cell impedance compared to the baseline electrolyte, improves the coulombic efficiency and reduces the voltage drop during storage, but it leads to significant gas generation^38^. Hall *et al*.^43^ demonstrated that a PES/DTD blend electrolyte generates different SEI formation from that produced with PES or DTD alone. DTD reacts with the reduction product of PES to form a new SEI film instead of undergoing direct reduction; however, its performance was not examined in the published paper^43^. MMDS has been reported as a multifunction additive suitable for a wide temperature range^45^ because it not only leads to a lower SEI impedance and reduces gas evolution but also passivates the CEI at elevated temperatures^46^.

It is difficult to develop electrolytes containing only one additive to obtain satisfactory electrochemical properties in all aspects, because single-additive electrolytes have some disadvantages. Blended salts are one option for use to enhance the comprehensive performance^47–51^. Lithium hexafluorophosphate (LiPF_6_) is an indispensable salt, which is beneficial due to its good solubility, high ionic conductivity and high ionic dissoc iation, but it has poor thermal stability and is easy to hydrolyse to generate HF, which is an important reason for the associated larger deterioration of performance^52^. Other salt-type electrolyte additives, such as lithium bis(oxalate)borate (LiBOB)^47,53^, lithium difluoro(oxalato)borate (LiDFOB)^54–56^, lithium bis(fluorosulfonyl)imide (LiFSI)^57,58^, lithium bis(trifluoromethylsulfonyl)imide (LiTFSI)^59^, and lithium difluorophosphate (LiDFP)^60–65^, could be partially substituted for LiPF_6_. It has been reported that the salt-type electrolyte additives LiFSI and LiBOB have a synergistic effect in enhancing battery performance^66^. Electrolytes with blended additives could directly and effectively improve the comprehensive performance of batteries by taking advantage of the synergistic effect of various substances. The Wang and Dahn^67^ group developed two series of electrolyte mixtures called PES222 (2 wt% PES + 2 wt% TTSPi + 2 wt% DTD) and PES211 (2 wt% PES + 1 wt% TTSPi + 1 wt% MMDS), which were added to the control electrolyte (1 M LiPF_6_, 3:7 w-w EC:EMC) in an NCM/C cell^68^. With 2% VC + 1% DTD + 0.5% TTSP + 0.5% TTSPi, all electrolyte systems showed significant improvement in electrochemical performance compared to the baseline electrolyte.

**Figure 1:**
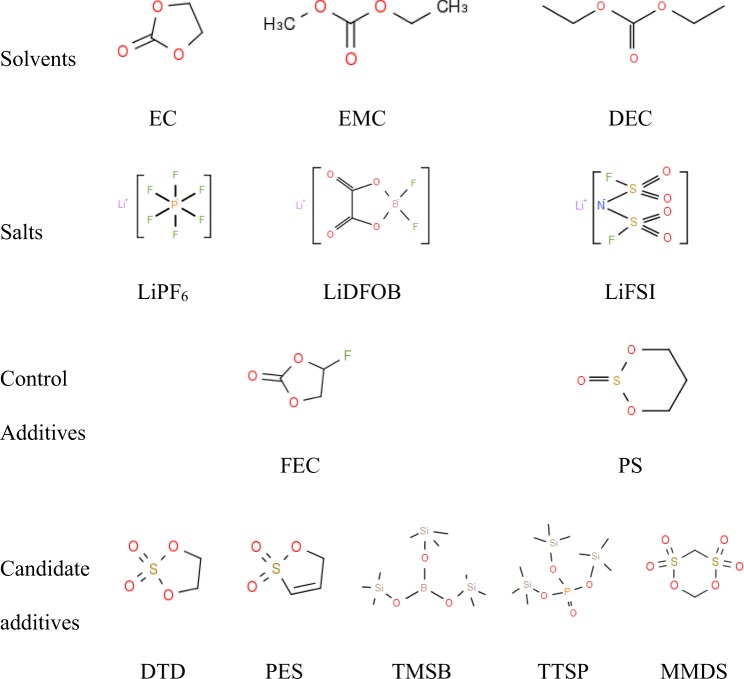
Structures of the solvents, salts and additives.

The development of electrolyte systems for high-energy density Li-ion cells that are compatible with nickel-rich cathodes and silicon-based anodes is still a great challenge. Hence, double difficulties arise concerning stabilizing the high-voltage CEI and modifying the unstable SEI film, both of which must be overcome by using a tailored system with blend additives. Therefore, we included the salt LiPF_6_, the blended salt-type electrolyte additive LiDFOB + LiFSI, and the major film-forming additive blend PS/FEC in the EC/EMC/DEC solvents in this work. We abbreviated the control electrolyte 1.0 M LiPF_6_ + 3:5:2 w:w:w EC:EMC:DEC + 0.5 wt% LiDFOB + 2 wt% LiFSI + 2 wt% FEC + 1 wt% PS as the baseline electrolyte (BL). Other candidate additives (see Fig. 1), such as DTD, the PES/DTD blend, TMSB, TTSP, and MMDS, whose advantages and disadvantages are shown in Table 1, were respectively added to the baseline electrolyte. The behaviours of obtained functional electrolytes were respectively characterized by cyclic voltammetry (CV), electrochemical impedance spectroscopy (EIS) and X-ray photoelectron spectroscopy (XPS) in positive and negative half-cells, as well as in full cells. Finally, the comprehensive performances of pouch cells, assessed through attributes including such as the beginning-of-life, charge-discharge cycle and storage performances, were examined for application.

**Table 1:** The advantages and disadvantages of candidate additives.

Additives	Advantage	Disadvantage	Reference
DTD	Dramatically enhances the cycle and storage performance	Instability and color during storage Large gas generation during formation	^36, 38^
PES	Inhibits gas generation at elevated temperatures; Reduces the transformation of rock-salt-type surfaces	impedance of the growth of the negative electrode by its oxidation product migrated from the positive electrode	^43^
TMSB	Inhibits the surface of the positive electrode and stabilizes the CEI	Hardly reductive (related to EC)	^11, 86^
TTSP	Produces an HF scavenging effect, protects the CEI	Hardly reductive (related to EC) Reactive with LiPF_6_, sometimes showing disadvantages	^11, 67, 86^
MMDS	Reduces the impedance growth Modifies the CEI, leading to less parasitic reactions Shows excellent life performance Reduces the gas evolution	Instability during storage	^73^

## Experimental Section

### Chemical

All electrolytes were prepared in a low-dew point (<−50°) lab. The control electrolyte used was 1.0 M LiPF_6_ (BASF, purity 99.94%, water content 14 ppm) in ethylene carbonate (EC)/ethyl methyl carbonate (EMC)/diethyl carbonate (DEC) (3/5/2 by weight, from BASF, water content <20 ppm). The control additives used in the experiment were lithium oxalyldifluoro borate (LiDFOB, Zhengzhou ALFA, 99.98%); lithium bis(fluorosulfonyl) imide (LiFSI, Suzhou Fortek, >99.9%); fluoroethylene carbonate (FEC, BASF, >99.95%); and propylene sulfite (PS, Aladdin, 99.5%), with the listed additives all being ingredients in the BL. In addition, several candidate additives were added to the BL, including 1,3,2-dioxathiolane 2,2-dioxide (DTD, Aldrich, 98%); prop-1-ene-1,3-sultone (PES, Aladdin, 98%); tris(trimethylsilyl) borate (TMSB, Aladdin, >98%); tris(trimethylsilyl) phosphate (TTSP, Aladdin, >97%); and methylene methanedisulfonate(MMDS, Aladdin, 98.70%). The baseline electrolyte (BL) was 1.0 M LiPF_6_ + 3:5:2 w-w:w EC:EMC:DEC + 0.5 wt% LiDFOB + 2 wt% LiFSI + 2 wt% FEC + 1 wt% PS. 1.5 wt% DTD, 1 wt% DTD + 0.5 wt% PES, 0.5 wt% TMSB, 0.5 wt% TTSP, and 0.5 wt% MMDS were added to the BL to form five formulations, respectively.

### Pouch cells

Experimental 4.4 Ah NMC811/ SiOx -based pouch cells were assembled, with the positive electrodes consisting of 97.6 wt% NMC811 material (KY18; Shanshan Technology; 1 C gravimetric capacity ≈ 186 mAh/g), 0.6 wt% CNTs (FT117-44; Cnano Technology, China) and 1.8 wt% PVDF (Solf 5130; Solvay, USA) and the negative electrodes consisting of 95.84 wt% SiO_*x*_/C/Gr composite material (S420-A; BTR, China; 1 C gravimetric capacity is 420 mAh/g), 1.3 wt% CMC (DAICEL 2200; Daicel FineChem Ltd, Japan), 1 wt% carbon black (Super-P Li; IMERY, Switzerland), 0.06 wt% SWCNTs (TUBALL BATT H_2_O; OCSAl, Russia) and 1.8 wt% SBR (AL-3001; A&L CO. LTD, Japan). In addition, a separator was included (ND16T40; SMCORP, China; 20 μm ceramic-coated PP/PE/PP films), which is listed in Table 2.

**Table 2:** Electrode composition and parameters of the pouch cells.

Properties	Positive electrode	Negative electrode
Composition	97.45 wt% NMC811 0.6 wt% CNTs slurry (CNTs:dispersant:solvent = 4 wt%:1 wt%:95 wt%) 1.8 wt% PVDF	95.72 wt% Si-based composite 1.3 wt% CMC 1 wt% Carbon black 0.06 wt% CNTs slurry (CNTs:dispersant:solvent = 0.2 wt%:0.4 wt%:99.4 wt%) 1.8 wt% SBR
Processing solvent	NMP	Water
Average areal capacity	3.9 mAh cm^−2^	3.58 mAh cm^−2^
Electrode loading	44 mg cm^−2^	23 mg cm^−2^
Current collector	Aluminium (16 μm)	Copper (8 μm)
Porosity	24.7%	26.3%

The dried pouch cells were filled with 15 g (approximately 3.4 g/Ah) electrolyte and then heated under vacuum for 30 s After sealing, the cells were allowed to rest for 24 h at 25 °C to ensure complete wetting of the electrodes and separator with the electrolyte. After constant-current 0.44 A (0.1 C) charging for 1 h to 3.4 V and constant-current 0.88 A (0.2 C) charging for 2.5 h to 3.7 V at constant temperature (35 °C) and external pressure (650 kPa), the formatted pouch cells were degassed in an argon-filled glove box.

### Coin cells containing fresh electrodes and harvested electrodes

The areas of the positive and negative electrodes are the same (1.54 cm^2^) for CR2032 coin cells. The fresh electrodes were dried in a vacuum oven at 100 °C for at least 3 h before the experiments and then transferred to a glove box with an argon atmosphere for fabrication. The working elect rodes (Si-based negative electrodes or NCM811 positive electrodes, 15 mm diameter), the counter electrodes (fresh lithium plate), the separator (20 μm PP/PE/PP ceramic-coated separator, 16 mm diameter), and all the coin cells were filled with 25 μL electrolyte solution. The assembled coin cells were rested for 8 h at room temperature to ensure that the electrolytes completely infiltrated the electrodes and separator before use.

For the coin cells containing harvested electrodes, after 190 cycles, the NCM811-SiO_*x*_/C/Gr pouch cells were discharged to 0% SOC and then transferred to a glove box with an Ar atmosphere to be disassembled. The harvested electrodes were washed with DMC (dimethyl carbonate, >98%) to remove excess electrolytes and dried in the glove box for 8 h. Some of the negative electrodes were used as working electrodes. Lithium plates were used as counter electrodes and reference electrodes, and then the cells were assembled following the same procedures used with the fresh electrodes.

### Performance test

The BOL performance was examined via the following procedure. The cells were charged/discharged between 2.5 V and 4.2 V with 1.32 A (0.3 C/0.3 C) for three cycles and then tested at 1.32 A (0.3 C) for charge and 4.4 A (1 C) for discharge across three cycles. The capacity of the last cycle of 1 C discharge was defined as the initial capacity of the cells. The impedance measurement was performed on the cells at 50% SOC and the frequency of 1 kHz on an internal resistance tester (HIOKI BT3562, Japan).

The cycle performance of the cells containing different electrolytes was examined with a charge/discharge current of 4.4 A (1 C/1 C) between 2.5 V and 4.2 V. A constant-voltage charge step at 4.2 V was performed until the current declined to 0.44 A (1 C).

The storage performance of the fully charged cells was tested at 55 ± 2 °C for 7 days. Then, the cells were tested with a charge current of 1.32 A (0.3 C) and a discharge current of 4.4 A (1 C) between 2.5 V and 4.2 V for three cycles to calculate the capacity retention and capacity recovery.

The charge-discharge experiments were all performed on a high-precision battery test system (Neware CT4008-5V20A-A, China) at 25 ± 2 °C, controlled by a climatic chamber (DGBELL BTT-80B-3, China).

### CV measurement

Cyclic voltammetry (CV) of the SiO_x_/C/Gr-Li half-cells and NCM811-Li half-cells was carried out on an electrochemical workstation (Biologic VMP-3, France) at 25 ± 2 °C. For the CV of SiO_*x*_/C-Li half cells, the scan rate was set to 0.05 mV/s from 0.005 V to 1.8 V versus Li/Li^+^, and the sweep was two turns. For the CV of NCM811-Li half cells, the CV was cycled only once at a scan rate of 0.05 mV/s from 3.0 V and 4.3 V.

### EIS measurement

The coin cells were first charged and discharged twice at 0.1 C/0.1 C, and then electrochemical impedance spectra (EIS) were obtained from 10 kHz to 10 mHz with an amplitude of 10 mV at 25 ± 2 °C using an electrochemical workstation (Biologic VMP-3, France).

### XPS measurement

The pouch cells were disassembled after 190 cycles to obtain the harvested positive and negative electrodes. Before X-ray photoelectron spectroscopy (XPS) testing, the harvested electrodes were rinsed with DMC to remove electrolyte residue and then dried overnight at room temperature. The negative electrodes were harvested from cycled coin cells following the same procedure. For comparison, fresh positive and negative electrodes were tested at the same time.

The experiment was carried out on the Thermo Escalab 250Xi X-ray diffractometer. The samples were transferred to the ultra-high vacuum chamber of the XPS system within l min to avoid surface changes of electrodes from exposure to air. A single-colour Al Kα source (hν = 1486.6 eV) was used to test the energy with a 10 mA filament current and a 40 eV filament voltage source. To compensate for the charging of the sample, a charge neutralizer was used. The measurement was performed by applying energy at 40 eV at an emission angle of 0°; a measurement time of 600 s was used with a lateral resolution of 3 μm, and the pressure in the analysis chamber of 10–7 Pa. The measurement data was fitted using Avantage software, and the fitting method was described with reference to other literature^69,70^.

## Results and Discussion

### Influence of electrolyte additives on the electrolyte stability

**Figure 2:**
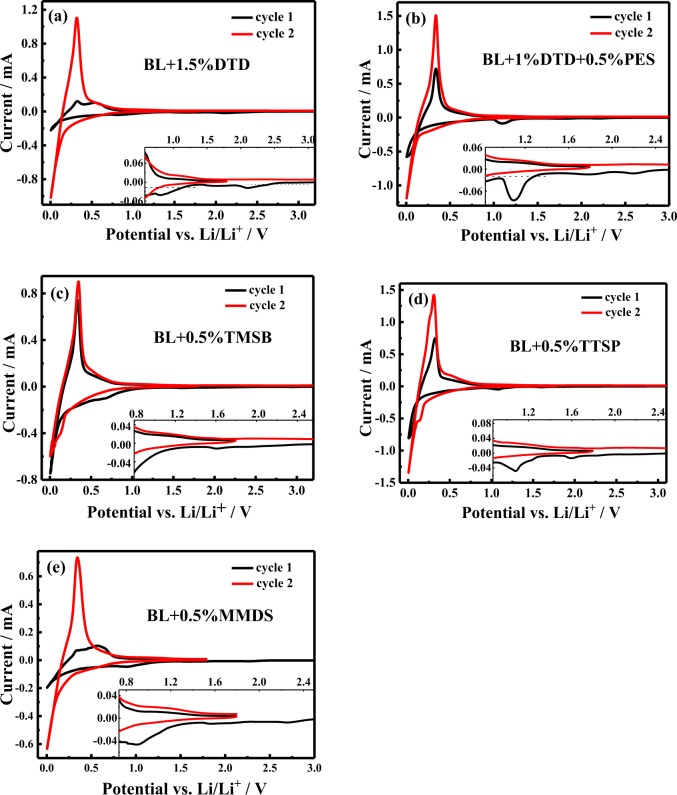
Cyclic voltammograms of SiO_*x*_/C/Gr-Li half-cells with electrolytes: (a) BL+1.5% DTD (b) BL+1.5% DTD+0.5% PES, (c) BL+0.5% TMSB, (d) BL+0.5% TTSP, and (e) BL+0.5% MMDS. Electrochemical window: 0.005−1.8 V vs Li/Li^+^; scan rate: 0.05 mV • s^-1^.

As seen from Table 3, additives such as LiDFOB, TMSB, TTSP show distinguishable reduction potentials compared to those of other additives, which indicates similar potentials between 1.0 V and 1.3 V. Figure 2 shows the CV curve of SiO_*x*_/C/Gr-Li half-cells containing electrolytes, and the reduction potentials of the peaks are listed and classified in Table S2. First, there are three reduction peaks on the curves of cyclic voltammetry for all electrolytes except BL + TMSB (two peaks), which complicate the analysis because some additives show similar reduction potentials between 0.8 V and 1.1 V. All the electrolytes show an obvious reduction peak at 1.6~1.8 V, possibly ascribed to the reduction of LiDFOB. However, the broadened peaks at 0.8~1.1 V shown for all electrolytes (except for BL + TMSB) and the special peak at 0.66 V for BL + TMSB are probably attributed to the reduction of LiFSI, the additives (FEC or PS) in the BL and each candidate additive itself. The peaks are broadened by the reduction peaks of each additive, so that they are hardly distinguishable. Furthermore, the special peak at 1.23 V for BL + TTSP is unique, which might be due to the reduction of FEC and is separated from the reduction peaks at 1.07 V. Finally, it is interesting that all the electrolytes with S-containing candidate additives show unique peaks at approximately 2.0 V~2.3 V, which cannot be attributed to any additives, salts or solvents. In our opinion, the peaks might be produced from a parasitic reaction caused by residual water, which is characteristic of all electrolytes with S-containing additives but is prevented in electrolytes with Si-containing additives.

**Table 3:** The reduction of reported additives.

Additives	Reduction potential (vs Li/Li^+^)	Reference
LiFSI	1.0 V (1:1 EC: DEC)	^58^
LiDFOB	1.60 V (1 M LiPF_6_ + 3:7 v:v FEC:EMC)	^87^
FEC	1.30 V (1 M LiPF_6_ + 1:1 w-w EC:DEC)	^88^
PS	~1.0 V (1 M LiPF_6_ + 3:7 w-w EC:EMC)	^89^
DTD	~1.25 V (1 M LiPF_6_ + 3:7 v:v EC:EMC) ~1.3 V (1 M LiPF_6_ + 3:7 v:v EC:EMC)	^38, 43^
PES	~1.15 V (1 M LiPF_6_ + 3:7 v:v EC:EMC)	^43^
TTSP	lower than EC (<~0.6 V, DFT), difficult to reduce	^86^
TMSB	lower than EC (<~0.6 V, DFT), difficult to reduce	^86^
MMDS	~1.2 V (1 M LiPF_6_ + 1:2 v:v EC:DEC)	^46^

### Impedance analysis

**Figure 3:**
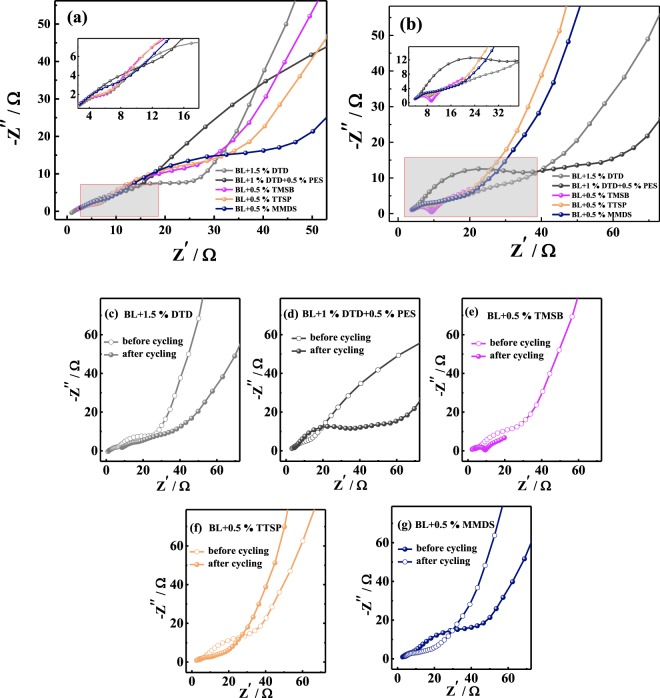
EIS diagram of SiO_*x*_/C/Gr-Li half-cells with different electrolytes (a) before and (b) after 50 cycles; (c) ~ (g) are comparisons of each electrolytes before and after cycling. (Frequency range: 0.1 Hz~10 kHz; amplitude: 5 mV).

As far as we know, the initial SEI impedance, which is shown in the first semicircle of the Nyquist diagram, can be calculated by curve fitting. Figure 3 shows the EIS plot of SiO_*x*_/C/Gr-Li half-cells containing different electrolytes before and after 50 cycles. Subsequently, the SEI impedance of the negative half-cells before and after 50 cycles and the impedance of harvested negative half-cells after 190 cycles were determined and are shown in Table S3. The initial impedance values shown in Table S3 follow the order of BL + DTD (3.57 Ω) < BL + MMDS (4.06 Ω) < BL + DTD + PES (4.82 Ω) < BL + TTSP (6.45 Ω) < BL + TMSB (6.75 Ω). The electrolytes with S-containing additives show lower initial impedances than do those with Si-containing additives, because reduction products such as Li_2_SO_3_ and Li_2_SO_4_ are derived from open-ring reactions, reducing the SEI impedance^38,71–73^. However, as seen from Fig. 3(b–g) and Table S3, the SEI impedance growth after 50 cycles followed the order of BL + TTSP (38%) < BL + TMSB (44%) < BL + MMDS (63%) < BL + DTD (69%) < BL + DTD + PES (104%). Therefore, the Si-containing additives showed an advantage in terms of the reduction of the SEI impedance increase in half-cells compared to the S-containing additives.

**Figure 4:**
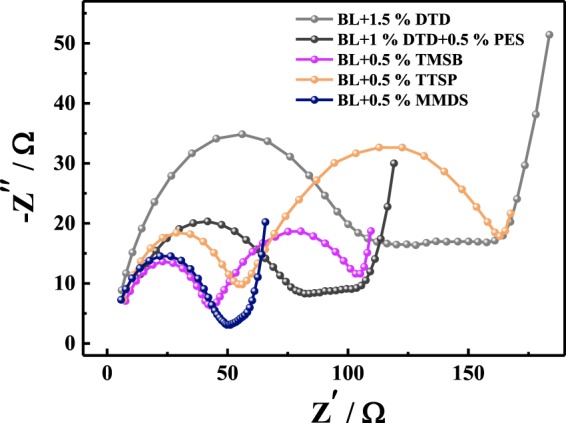
EIS diagram of harvested SiO_*x*_/C/Gr-Li half-cells containing different electrolytes. The negative electrodes were harvested after 190 cycles. (Frequency range: 0.1 Hz~10 kHz; amplitude: 5 mV.

To ascertain the mechanism of reducing the impedance growth, the SEI impedances of negative electrodes harvested from the 190-cycled full cells were checked. Figure 4 shows the EIS diagrams of harvested SiO_*x*_/C/Gr-Li half- cells containing different electrolytes evaluated according to those of the negative electrodes of the full cells har vested after 190 cycles. The SEI impedance followed the order of BL + TMSB (37.3 Ω) < BL + MMDS (45.3 Ω) < BL + TTSP (54.3 Ω) < BL + DTD + PES (63.5 Ω) < BL + DTD (125.4 Ω). The harvested negative electrodes had a large SEI impedance growth compared to that of the initial state, which means that the SEI formation on the negative electrode and the composition are largely affected by the positive electrodes in the full cell. James reported that chemical and electrochemical crosstalk may occur inside the cell, and the reduction product of the electrolyte additive on the positive electrode may also be transferred to the negative electrode, so the resulting properties are different from those of the half cell^74^. As a sulfur-containing additive, the impedance of the MMDS is very small after cycling, which is in contrast to the case for DTD and PES, which might be affected by crosstalk between the positive and negative electrodes, leading to dissimilar behaviours from those of the half cell^74^. As a sulfur-containing additive, the impedance of the MMDS is very small after cycling, which is in contrast to the case for DTD and PES, which might be affected by crosstalk between the positive and negative electrodes, leading to dissimilar behaviours in the half-cells. For silicon-containing additives, the impedance of BL + TTSP is slightly larger than that of BL + TMSB, but the impedances are both smaller than those of DTD and PES. It is reasonable to speculate that damage to the SEI and an impedance increase of the positive electrode from eluted ions are the main causes of the increase of the full-cell impedance, and TTSP/TMSB can suppress this attenuation trend in the positive electrode.

### XPS analysis

The overall performance of cell is significantly influenced by the electrode/electrolyte interfaces, which are comprise of chemical and electrochemical decompositions^75^. Consequently, the XPS analysis was carried out to ascertain the compositions and verify the possible effect of additives on the performance of cathode and anode.

**Figure 5:**
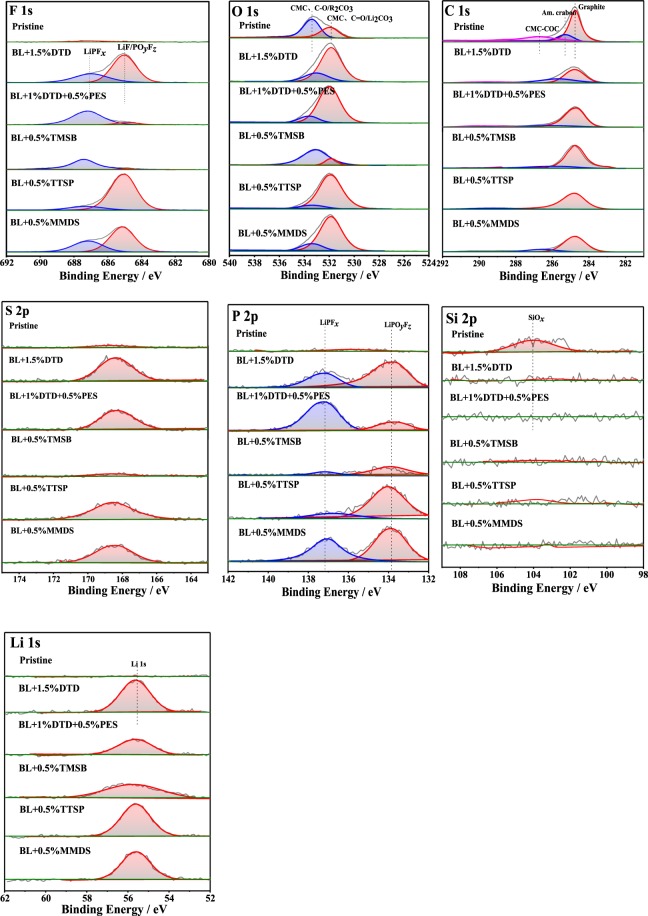
XPS spectra (F 1s, O 1s, C 1s, S 2p, P 2p, Si 2p, Li 1s) of the SiO_*x*_ -based anodes after 50 cycles from the half-cells with different electrolytes.

Figure 5 shows the F 1s, O 1s, C1s, S 2p, P 2p, Si 2p, and Li 1s spectra of the surfaces of negative electrodes harvested from full cells after 50 cycles and half cells containing different electrolytes. The average atomic concentration percentages (at %) of the surface layer are listed in Table S4.

In the F 1s spectra of all cycled electrodes, there are two peaks at ≈685.1 eV and ≈687.7 eV, which is belong to the formation of LiF/PO_*y*_F_*z*_ species and LiPF_*x*_ species respectively^74^. Generally, the content at % of the LiPF_*x*_ resulting from BL + S-containing additives is much great than that from Si-containing additives, and the peaks of LiF/LiPO_*y*_F_*z*_ are absent from the BL + DTD + PES and BL + TMSB spectra.

In the spectra of P 2p, the electrolytes containing BL + DTD + PES and BL + TMSB show low LiPO_*y*_F_*z*_ contents (0.31% and 0.49%), while those of the electrolytes DTD, TTSP and MMDS are significantly higher (1.07%, 1.23%, and 1.18%). Furthermore, the relatively small content of LiPO_*y*_F_*z*_ compared to the content of LiF/LiPO_*y*_F_*z*_ for BL + DTD, BL + TTSP and BL + MMDS, for which the data are shown in Table S4, indicate that the F-containing products in the negative electrode SEI are likely to be mostly LiF.

There is only a broad peak at ≈533.4 eV for the pristine electrode in the O 1 s spectra, contribute to the small amounts of oxygen arise from the surface of graphite and CMC binder^70,76^. As for the cycled electrodes, the intensity of peaks decreased as the CEI film formed. Additionally, the peaks at ≈532 eV increases significantly which is due to Li_2_CO_3_ formation. However, the asymmetric peak was assigned to the mixtures of (LiRCO_3_), R_2_CO_3_, CMC, and Li_2_CO_3_ species, leading to hardly identification^77^.

Unfortunately, the same issue is present in the C 1 s spectra. The broadened peak prevents exact differentiation between the different species in the C 1 s spectra. From the peak at ~284.7 eV from the electrolytes, it can be seen that the peak of graphite is reduced, but the peak value may also be affected by other products, so the thickness of the SEI cannot be accurately evaluated^70^. The peaks of C 1 s at 282.2 eV are absent for the pristine electrode and the aged electrodes, indicating that LiC_*x*_ was not formed and that there was no loss of irreversible active lithium.

In the spectrum of S 2p, each electrode coupled with BL + DTD, BL + DTD + PES and BL + MMDS produced a peak at ~169.4 eV, which is due to the reduction of PS in the BL and the reduction of sulfur-containing candidate additives in the anode^38^. Moreover, there is a lower reduction peak of PS shown in the spectrum of BL + TTSP, but no peaks were formed on the curve of BL + TMSB, indicating that the reduction of PS is suppressed by TMSB.

It can be observed from the Si 2p spectra that the Si element is found in the pristine electrode but can’t be detected in the aged electrodes. This is because during the long-term cycles, the continuously formed SEI is thick enough to cover Si, leading to the loss of signs of the Si 2p. It is worthwhile to note that there are no peaks on the curves of BL + TTSP and BL + TMSB, meaning that the Si-containing additives could not participate in SEI formation on the anode in these cases.

**Figure 6:**
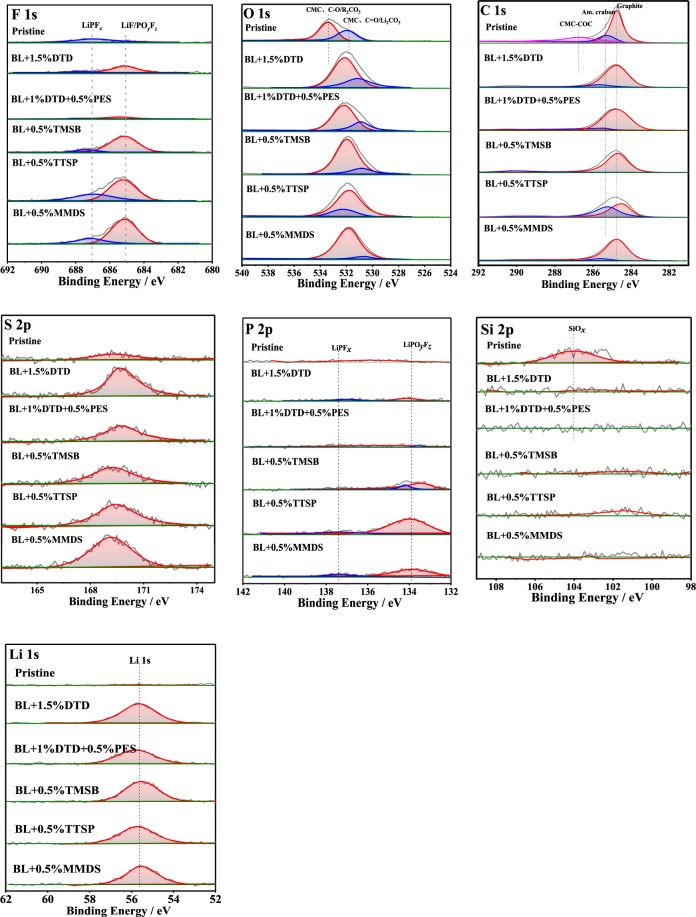
XPS spectra (F 1s, O 1s, C 1s, S 2p, P 2p, Si 2p, Li 1s) of the pristine and harvested SiO_*x*_ -based anodes from the full cells with different electrolytes which are full-discharged after 190 cycles.

Figure 6 shows the F 1s, O 1s, C1s, S 2p, P 2p, Si 2p, and Li 1s spectra of the surfaces of negative electrodes harvested from full cells after 190 cycles. The average atomic concentration percentages (at. %) of the surface layer are listed in Table S5 in the supporting information.

The conclusion obtained for the full-cell is similar to that for the half-cell. The composition of the SEI is mainly inorganic, consisting of the reduction/decomposition products of the additives and the lithium salts. The spectra of the full cells and half-cells are also different. On the spectra of the negative electrodes harvested from the half-cells, the positions of the peaks of P 2p in BL + TMSB are observed to be significantly misaligned with respect to those of the other systems. In addition, BL + DTD and BL + DTD + PES show higher F 1s and P 2p contents in the half-cells, but these substances are hardly detected in the full cells. It is possible that in both electrolyte systems, the decomposition products of LiPF_6_ mainly accumulate on the surface of the positive electrode.

**Figure 7:**
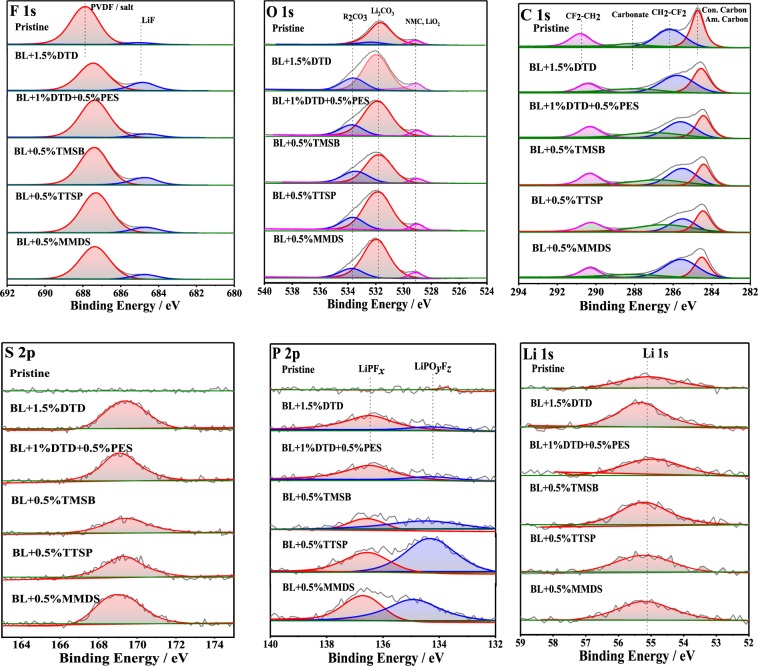
XPS spectra (F 1s, O 1s, C 1s, S 2p, P 2p, Li 1s) of pristine and harvested NCM811 taken from the full cells with different electrolytes after 190 cycles.

Figure 7 shows the F 1s, O 1s, C 1s, P 2p, Li 1s and S 2p spectra of the positive electrode surfaces of the full cells containing different electrolytes after 190 cycles. The average atomic concentration percentages (at %) of the surface layer are listed in Table S6.

It can be seen from the spectrum of F 1s that the peak at ≈687.8 eV is ascribed to the C-F bond in PVDF binder^69^. Additionally, there are a small peaks at ≈685 eV, is attributed to the LiF at CEI, which possibly arise from the parasitic reactions of PVDF^78,79^.

The O 1s spectrum shows a peaks at ≈531.6 eV and a small shoulder peak at ≈532.3 eV, which are possibly assigned to the Li2CO3, low-coordinated oxygen atoms and other surface impurity^21,80^. Additionally, the peaks at ≈529.2 eV is belong to the O_2_ anion in NCM811^81^.

The C 1s spectrum contains multiple contributions—in particular, from the CF_2_ (CF_2_-CH_2_; at ≈290.8 eV) and CH_2_ (CH_2_-CF_2_; at ≈286.1 eV) groups of PVDF, respectively—and an additional large peak at ≈284.7 eV that results from the C–H and C–C groups of the conductive carbon substance^82,83^. As expected, no peaks are present in the P 2p spectra of the pristine electrode, but it produces a certain Li peak at ≈55.1 eV.

Compared with the original electrode, the harvested electrodes showed a significant decrease in the F 1 s peak at ≈687.8 eV and the C 1s peak at ≈284.7 eV, indicating that a dense surface film was formed on the positive electrode, thus reducing the peak intensity of the cathode material. The peak intensity of the O 1 s at 529.2 eV (oxygen in NCM811 active material) indicates that the thicknesses of the oxide films formed by the electrolytes BL + DTD + PES, BL + TTSP, and BL + MMDS are approximately the same, and this is because, as can be seen from Table S6, the atomic percentages of these electrolytes are similar (1.13%~1.27%). It can be seen from the spectrum of F 1 s and the relative atomic percentage table that the contents of LiF, which has been identified as a common decomposition product of LiPF_6_, are significantly increased. However, the cumulative F concentrations of the five electrolytes are about the same.

From the spectrum of O 1 s, the peak intensities of the electrolytes at ~531.6 eV were significantly increased, indicating that the electrolyte solvent and lithium salt were decomposed. From the peak at ≈532.3 eV in the O 1 s spectrum, the content of alkyl carbonate was significantly increased compared to that in the pristine electrode. For the pristine electrode, the value of the alkyl carbonate was 1.06%, and those of the electrolytes containing DTD, DTD + PES, TMSB, TTSP, and MMDS were respectively approximately 4.06%, 3.42%, 3.97%, 4.11%, and 3%. It is easy to observe that the electrolyte containing TTSP shows the highest value of alkyl carbonate, indicating the CEI modification ability of TTSP. The significant increase of the average atomic concentration of carbonates according to C 1 s and P 2p spectra can also demonstrate the modification of CEI by additives.

In the P 2p spectrum of the electrolyte BL + TTSP, the content of LiPO_*y*_F_*z*_ (1.09%) was much higher than those of the other electrolytes, indicating that TTSP is involved in the formation of the CEI^84^. In addition, the peaks of PVDF and LiPO_*y*_F_*z*_ in the F 1 s spectrum are overlapped, further affecting the identification of the reactants. As far as we know, LiPO_*y*_F_*z*_ has favourable effects on the stability of the CEI and the cycle performance of the cell. Therefore, the electrolyte containing TTSP could enhance the overall performance of NCM811 electrodes.

It can be observed from the spectrum of S 2p that the harvested electrodes showed a distinct peak at 169.4 eV. The peaks were shown in the spectra of TMSB and TTSP, indicating that the PS was oxidized as reported. However, peaks of larger intensities are observed in the spectra of DTD, PES, and MMDS, proving that DTD, PES, and MMDS can react on the positive electrode of the full-cell and that they may be oxidized on the surface of the positive electrode or participate in chemical reactions to form the CEI. Finally, some of the electrode materials (the conductive agent, PVDF, and NCM811) could still be detected because the CEI formed by the positive electrode was sufficiently thin (<5 nm)^85^.

### The BOL performance

**Figure 8:**
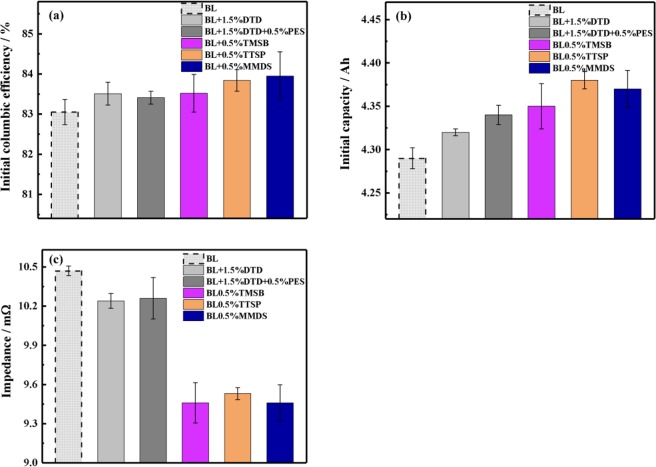
Influence of the addition of 1.5 wt% DTD, 1 wt% DTD + 0.5 wt% PES, 0.5% wt% TMSB, 0.5 wt% TTSP and 0.5 wt% MMDS into the BL electrolyte on the BOL performance of fresh cells. Depicted are (a) the initial columbic efficiency (ICE); (b) the initial discharge capacity; and (c) the initial impedance (1 kHz).

The electrolyte system was first evaluated according to the beginning-of-life (BOL) performance of the fresh cells, which includes the initial columbic efficiency (ICE), the initial discharge capacity, and the initial impedance. It can be seen from Fig. 8 that cells containing various electrolytes including base electrolyte exhibited varying differences in the BOL performance, and the data are shown in Table S7. From Fig. 8(a), the initial columbic efficiencies of the fresh cells containing the electrolytes followed the order of MMDS (83.95%) > TTSP (83.84%) > TMSB (83.52%) ≈ DTD (83.51%) > PES + DTD (83.41%) > BL (83.19%). However, they fell within the range of 83%~84% (from Table S7), which indicates slight difference among all electrolytes. From Fig. 8(b), the initial discharge capacity followed approximately the same trend as that of the initial columbic efficiency. It followed the order of TTSP (4.38 Ah) > MMDS (4.37 Ah) > TMSB (4.35 Ah) > DTD + PES (4.34 Ah) > DTD (4.32 Ah) > BL (4.29Ah). Among the evaluated cells, the cell containing BL + TTSP showed the largest capacity at 4.38 Ah, which was approximately 1% more than the next closest capacity of 4.32 Ah of the cell containing BL + DTD. Moreover, according to Fig. 8(c), the alternating-current impedance of the cells followed the order of TTSP (9.46 mΩ) ≈MMDS (9.46 mΩ) < TMSB (9.53 mΩ) < DTD + PES (10.24 mΩ) ≈ DTD (10.26 mΩ) < BL (10.47 mΩ), which is more or less in contrast to the trend of the initial capacity, implying the correlation between impedance and capacity. Obviously, either S-containing additives or Si-containing additives both improve the BOL performance by comparing to the base electrolyte.

In summary, the pouch cells with Si-containing additives TTSP and TMSB showed less impedance and much more capacity than did cells with S-containing additives, indicating the advantages of Si-containing additives over S-containing additives in BL electrolyte systems. For the Si-containing additives, the BOL performance of TTSP is 83.84%, 4.38 Ah and 9.53 mΩ, while the values for TMSB are 83.5%, 4.35 Ah and 9.46 mΩ. TTSP shows a slightly worse impedance and slightly larger initial efficiency but a larger capacity than does TMSB (4.35 Ah), and the EIS measurements in Fig. 4 show the same trend concerning the comparison of the initial impedance (1 kHz) values, indicating that a better SEI film was formed by TTSP during the formation stage than by TMSB. In addition, for the S-containing additives, cells containing DTD electrolytes showed less capacity than did those containing MMDS, possibly due to the much greater initial impedance of the cells induced by DTD electrolytes. Finally, we draw a conclusion that electrolytes including TMSB, TTSP and MMDS can suppress parasitic reactions and form a less lithium-consuming SEI layer to obtain a better BOL performance.

### Long-term cycling

**Figure 9:**
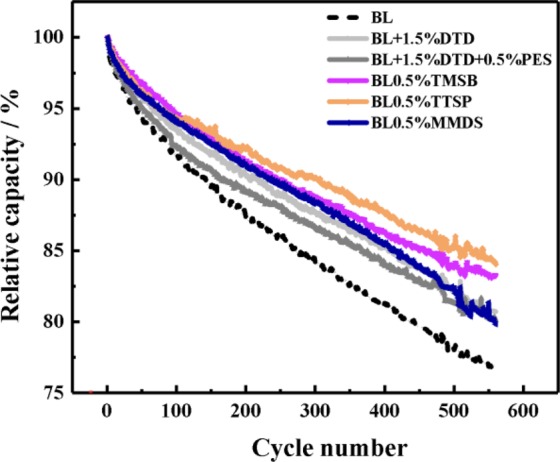
Influence of the addition of 1.5 wt% DTD, 1 wt% DTD + 0.5 wt% PES, 0.5% wt% TMSB, 0.5 wt% TTSP and 0.5 wt% MMDS into the BL electrolyte on the cycling behaviour of experimental pouch cells.

Figure 9 describes the long-term cycling behaviour of cells containing different electrolytes, which is used to evaluate the durability performance of the SEI film. Until the 560th cycle, the capacity retention of long-term cycles followed the order of TTSP (84.05%)å TMSB (83.24%)å DTD (80.72%)å DTD + PES (84.10%) > MMDS (79.84%) > BL (76.93%), for which the data are shown in Table S8. It is obvious that the capacity retention capabilities of Si-containing TTSP and TMSB are better than those of S-containing additives in improving cell long-term cycling performance, which turns out that the film-forming stability of the Si-containing additives is more suitable for NMC811/silicon-based cells. There is a unique behaviour of capacity fading for electrolytes containing MMDS, which yields larger performance deterioration than do the others. The cells containing MMDS show a larger capacity retention that do those with other S-containing additives before 300 cycles but less capacity retention than do those with other S-containing additives after 500 cycles. Moreover, the cells show the worst end-of-life (80% state-of-health) performance, at approximately 560 cycles. This behaviour indicates that MMDS causes a significant increase in the internal impedance of the cell, which is related to the decomposition from consumption on the positive electrode.

### Storage at elevated temperatures

**Figure 10:**
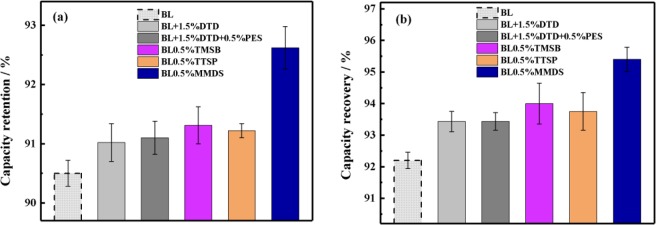
Influence of the addition of 1.5 wt% DTD, 1 wt% DTD + 0.5 wt% PES, 0.5% wt% TMSB, 0.5 wt% TTSP and 0.5 wt% MMDS into the BL electrolyte on the storage behaviour of pouch cells at elevated temperatures. Depicted are (a) the capacity retention and (b) the capacity recovery.

The parasitic reaction at the interface between the positive electrode and the electrolyte is the main factor in the degradation of the cell performance at elevated temperatures and high voltages. Figure 10 shows the storage performance of fully charged cells containing different electrolytes at 55 °C for 7 days. From Fig. 10(a,b), the capacity retention and recovery of the cells show the same trend, following the order of MMDS > TMSB > TTSP > DTD + PES≈DTD > BL. The corresponding data are shown in Table S9 and Fig. S1. In general, the Si-containing additives TMSB and TTSP show more capacity retention and recovery in the cells than do the S-containing additives DTD and DTD + PES. For the Si-containing additives, the capacity retention and recovery of TMSB were 91.31% and 94%, respectively, which are slightly better than those of TTSP, with values of 91.22% and 93.75%. For the S-containing additives, the capacity retention and recovery of DTD were 91% and 93.4%, respectively, while the cells containing DTD + PES showed identical capacity retention and recovery values, indicating that adding PES cannot enhance the storage performance of the cells. However, MMDS has prominent advantages over all other electrolytes in the BL electrolyte system. Its capacity retention and recovery were 92.6% and 95.4%, respectively. Taken together, the MMDS in the S-containing additives significantly improves the interface durability of the positive electrode at elevated temperatures.

## Conclusion

In this study, in the electrolyte system for high-energy density Li-ion cells, DTD, DTD + PES, TTSP, TMSB, and MMDS were added to the baseline electrolyte (BL = 1.0 M LiPF_6_ + 3:5:2 w:w:w EC: EMC: DEC + 0.5 wt% LiDFOB + 2 wt% LiFSI + 2 wt% FEC + 1 wt% PS), and the mixtures were systematically studied in coin-type half-cells and pouch cells through CV, EIS and XPS experiments. Furthermore, the critical electrochemical performances of all systems were examined.

In general, for S-containing additives, the distinct reduction products (LiSO_3_ and Li_2_SO_4_) formed on the Si-based anode exhibited lower SEI impedance than those of Si-containing additives, according to the EIS measurement of half cells. However, The Si-containing additives (X(OR)_3_ derivatives, x = P or B, and R = TMS (-SiMe_3_)) show better long-term durability performance and storage performance than S-containing additives (SO_3_-derivatives (DTD, MMDS) and SO_4_-derivatives (PES)), which was attributed to the function of protection of the Ni-rich cathodes by Si-containing additives. In other words, the performance of high-energy density cells combined with Ni-rich cathodes and silicon-based anodes might be predominantly determined by the CEI, not by the SEI.

Therefore, a worse performance in terms of long-term and elevated- temperature durability is expected for S-containing additives according to this hypothesis. In contrast to DTD and DTD + PES, the MMDS-containing electrolyte showed a better storage performance, even comparable to those of electrolytes with Si-containing additives. Additionally, among the Si-containing additives, TTSP shows a slightly better performance than does TMSB, indicating TTSP as the most powerful cathode-protective agent.

## Supplementary information

S-containing and Si-containing compounds as highly effective electrolyte additives for SiOx -based anodes/NCM 811 cathodes in lithium ion cells
