# Supplementary material for: S-containing and Si-containing compounds as highly effective electrolyte additives for SiOx -based anodes/NCM 811 cathodes in lithium ion cells
Source: Sci Rep. 2019 Oct 1;9:14108. doi: 10.1038/s41598-019-49568-1 (PMC6773705; doi:10.1038/s41598-019-49568-1)
Supplement: Supplementary file 1 — S-containing and Si-containing compounds as highly effective electrolyte additives for SiOx -based anodes/NCM 811 cathodes in lithium ion cells [file 41598_2019_49568_MOESM1_ESM.pdf]

S-containing and Si-containing compounds as highly effective electrolyte additives for SiOx -based anodes/NCM 811 cathodes in lithium ion cells

Fuqiang An[a,b], Hongliang Zhao[c], Weinan Zhou[c], Yonghong Ma[c], Ping Li[a]

[a]Beijing University of Science and Technology, No.30 Collage Road, Haidian District, Beijing, China
[b]Shanxi Changzheng Power Technology Co.,Ltd. Shanxi, China
[c]Idrivetech Automobile Co., Ltd. No. 2 Nanqi Road, ChangPing District, Beijing, China

Table S1. List of chemicals used and their purity, abbreviations, and suppliers

Abbreviation	Purities	Suppliers
$LiPF_6$	purity 99.94%,<br>water content 14 ppm	BASF
EC - ethylene carbonate	99.9%<br>water < 10 ppm	BASF
EMC - ethyl methyl carbonate	99.92%<br>water < 6 ppm	BASF
DEC - diethyl carbonate	99.92%<br>water < 6 ppm	BASF
LiODFB - Lithium oxalyldifluoro borate	99.98%	Sigma-Aldrich
LiFSI- Lithium bis(fluorosulfonyl) imide	> 99.9%	Sigma-Aldrich
PS - Propylene sulphite	99.5%	Aladdin
FEC - Fluoroethylene carbonate	>99.95%	BASF
DTD - 1,3,2-Dioxathiolane 2,2-dioxide	98%	Sigma-Aldrich
PES - prop-1-ene-1,3-sultone	98%	Aladdin
TMSB - Tris(trimethylsilyl) borate	>98%	Aladdin
TTSP - Tris(trimethylsilyl) phosphate	>97%	Aladdin
MMDS - methylene methanedisulfonate	98.7%	Aladdin

Table S2. The reduction potentials of each peak

	peak 1	peak 2	peak 3
BL+1.5% DTD	0.80 V (LiFSI, PS, FEC, DTD)	1.60 V (LiDFOB)	2.08 V (parasitic reaction caused by residual water)
BL+1% DTD+0.5% PES	1.08 V (LiFSI, PS, FEC, DTD, PES)	1.77 V (LiDFOB)	2.19 V (parasitic reaction caused by residual water)
BL+0.5% TMSB	0.66 V (LiFSI, PS, FEC, TMSB)	1.70 V (LiDFOB)	
BL+0.5% TTSP	1.07 V (LiFSI, PS, TTSP)	1.60 V (FEC)	1.66 V (LiDFOB)
BL+0.5% MMDS	0.90 V (LiFSI, PS, FEC, MMDS)	1.58 V (LiDFOB)	2.25 V (parasitic reaction caused by residual water)

Table S3. The SEI impedances of negative half-cells before and after 50 cycles, and the impedance of harvested negative half-cells after 190 cycles

	Negative electrodes in cycled half-cells			Negative electrodes harvested from cycled full-cells (Ω)
Electrolytes	Before (Ω)	After (Ω)	Change (%)	
BL+DTD	3.57	6.04	69%	125.4
BL+DTD+PES	4.82	9.83	104%	63.5
BL+TMSB	6.75	9.74	44%	37.3
BL+TTSP	6.45	8.93	38%	54.3
BL+MMDS	4.06	6.61	63%	45.3

Table S4. Mean atomic concentration percentages (at. %) of the surface layer (F 1s, Mn 2p, O 1s, C 1s, S 2p, P 2p, Si 2p and Li 1s) on pristine and harvested SiO_x/C/Gr electrodes taken from full-discharged half-cells with several electrolytes after 50 cycles

Region	Component	BE / eV	Pristine / at. %	BL+1.5% DTD / at. %	BL+1% DTD+0.5% PES / at. %	BL+0.5% TMSB / at. %	BL+0.5% TTSP / at. %	BL+0.5% MMDS / at. %
F 1s	LiF/ $LiPO_yF_z$	685.1	0	7.83	0.59	0.21	11.28	7.89
F 1s	$LiPF_x$	687.7	0.84	3.68	5.56	4.65	1.56	4.09
O 1s	CMC, C-O/ R_2CO_3	533.4	8.35	4.22	2.97	10.59	1.77	3.3
O 1s	CMC, C=O /Li_2CO_3	532	5.34	14.69	17.48	2.04	16.44	16.1
C 1s	Graphite	284.7	50.53	25.07	42.22	46.99	28.22	31.84
C 1s	Am. Carbon	285.3	13.31	13.9	4.98	11.77	10.37	7.88
C 1s	CMC-COC	286.7	18.98	3.91	2.5	0	1.53	1.52
S 2p	$-SO_3$, $-SO_4$	169.2	0.28	1.02	0.92	0.3	0.91	0.9
P 2p	$LiPO_yF_z$	135.9	0.22	1.07	0.31	0.49	1.23	1.18
P 2p	$LiPF_x$	137	0	0.49	1.11	0.18	0.26	0.9
Si 2p	SiC_x	103.4	0.43	0.29	0.49	0.33	0.27	0.43
Li 1s	Li 1s	55.7	0	23.52	15.16	22.32	23.61	20.85

Table S5. Mean atomic concentration percentages (at %) of the surface layer (F 1s, Mn 2p, O 1s, C 1s, S 2p, P 2p, Si 2p and Li 1s) of pristine and harvested SiO_x/C/Gr electrodes taken from full-discharged pouch cells with different electrolytes after 190 cycles

Region	Component	BE / eV	Pristine / at. %	BL+1.5% DTD / at. %	BL+1% DTD+0.5% PES / at. %	BL+0.5% TMSB / at. %	BL+0.5% TTSP / at. %	BL+0.5% MMDS / at. %
F 1s	LiF/ $LiPO_yF_z$	685.1	0	0.88	1	1.89	2.27	2.74
F 1s	$LiPF_x$	687.7	0.84	0.27	0.15	0.25	1.09	0.91
O 1s	CMC, C-O / R_2CO_3	533.4	8.35	13.85	12.73	16.78	14.86	16.59
O 1s	CMC, C=O /Li_2CO_3	532	5.34	5.54	4.55	3.41	3.97	1.58
C 1s	Graphite	284.7	50.53	46.65	51.98	45.01	29.06	32.92
C 1s	Am. Carbon	285.3	13.31	10,27	10.32	8.66	26.17	21.34
C 1s	CMC-COC	286.7	18.98	3.51	4.16	4.42	4.31	4.67
S 2p	$-SO_3$, $-SO_4$	169.2	0.28	0.53	0.41	0.39	0.59	0.7
P 2p	$LiPO_yF_z$	135.9	0.22	0.11	0.21	0.4	0.73	0.44
P 2p	$LiPF_x$	137	0	0.08	0	0	0.14	0.13
Si 2p	SiC_x	103.4	0.43	0	0	0	0	0
Li 1s	Li 1s	55.7	0	18.05	14.31	18.39	16.66	17.72

Table S6. Mean atomic concentration percentages (at %) of the surface layer (F 1s, Mn 2p, O 1s, C 1s, P 2p, S 2p and Li 1s) of pristine and harvested NMC811 electrodes taken from taken from full-discharged pouch cells with different electrolytes after 190 cycles

Region	Component	BE / eV	Pristine / at. %	BL+1.5% DTD / at. %	BL+1% DTD+0.5% PES / at. %	BL+0.5% TMSB / at. %	BL+0.5% TTSP / at. %	BL+0.5% MMDS / at. %
F 1s	PVDF / salt	687.8	25.19	16.6	20.42	20.96	18.45	20.49
F 1s	LiF	685	1.21	4.5	1.85	4.25	2.24	2.63
O 1s	NMC, LiO_2	529.2	0.94	2.62	1.13	0.84	1.33	1.27
O 1s	R_2CO_3	532.3	1.06	4.06	3.42	3.97	4.11	3
O 1s	Li_2CO_3	531.6	8.71	13.24	14.25	10.6	15.75	14.94
C 1s	Con. Carbon, Am. Carbon	284.7	21.22	15.53	13.39	20.36	21.88	11.9
C 1s	Carbonate	286.1	19.94	20.01	17.35	13.88	13.78	22.23
C 1s	CF_2-CH_2	290.8	10.75	6.18	8.3	8.64	6.97	6.7
C 1s	CH_2-CF_2	288.2	4.37	6.44	10.33	6.01	5.29	5.88
C 1s	PEO	286.4	0	0	0	0	0	0
S 2p	$-SO_3$, $-SO_4$	169.4	0	1.07	1.11	0.8	1.14	1.23
P 2p	$LiPO_yF_z$	134.2	0	0.19	0.3	0.55	1.09	0.8
P 2p	$LiPF_x$	136.5	0	0.53	0.46	0.26	0.61	0.68
Li 1s	Li 1s	55.1	5.9	9.21	7.33	8.57	6.77	7.79

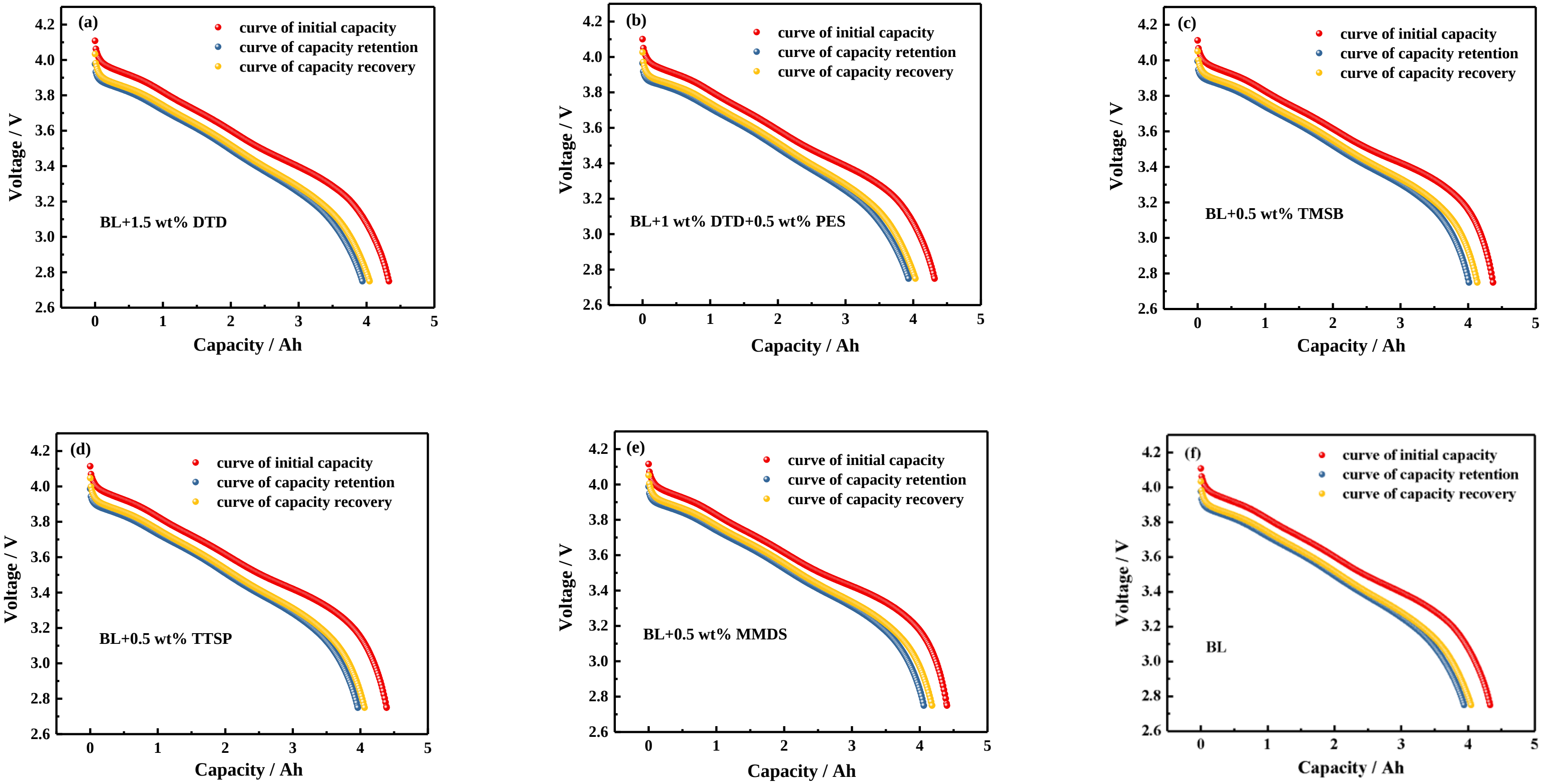

Figure S1(a)-(f) The charge-discharge curves of the initial capacity, verification of the capacity retention and the recovery of fully charged cells containing various electrolytes after storage at elevated temperatures.
